# Tumour-necrosis factor from the rabbit. IV. Purification and chemical characterization.

**DOI:** 10.1038/bjc.1980.253

**Published:** 1980-09

**Authors:** N. Matthews, H. C. Ryley, M. L. Neale

## Abstract

**Images:**


*Br. J. Cancer* (1980) **42**, 416

### TUMOUR-NECROSIS FACTOR FROM THE RABBIT. IV. PURIFICATION AND CHEMICAL CHARACTERIZATION

N. MATTHEWS, H. C. RYLEY AND M. L. NEALE

*From the Department of Medical Microbiology, Welsh National School of Medicine, Cardiff*

Received 28 April 1980 Accepted 19 June 1980

**Summary.—Serum from rabbits with BCG/endotoxin-induced shock is growth inhibitory or cytotoxic to a range of tumour cell lines.**

**The active component, tumour-necrosis factor (TNF), has been purified 1000-fold by sequential salt precipitation, ion-exchange chromatography and gel-filtration. TNF had a mol. wt of 67,000 on gradient PAGE and 39,000 on Ultrogel AcA44 gel-filtration. The isoelectric point was pH 5·1–5·2.**

**TNF was susceptible to the proteolytic enzyme pronase, but resistant to trypsin or papain. On isopycnic ultracentrifugation it had a buoyant density of 1·27, confirming that it is protein in nature, with little or no carbohydrate. This is also suggested by its failure to bind to a range of lectins.**

TUMOUR NECROSIS FACTOR (TNF) is a product of mononuclear phagocytes (Matthews, 1978) which is toxic *in vivo* and *in vitro* to some tumour cell lines (Carswell *et al.*, 1975; Matthews & Watkins, 1978). A potent source of TNF is the serum of animals with an endotoxic shock induced by i.v. injections, 2 weeks apart, of Bacillus Calmette Guerin (BCG) and endotoxin (Carswell *et al.*, 1975).

Mouse TNF has $\alpha$ electrophoretic mobility, a mol.wt of 150,000 as determined by gel filtration and is found in a glycoprotein-rich fraction of serum (Green *et al.*, 1976) Previously we have shown that rabbit TNF also has $\alpha$ electrophoretic mobility, but it is smaller than its mouse counterpart, with a mol. wt by gel-filtration of 40–50,000 (Matthews & Watkins, 1978). In this paper the physicochemical characteristics of rabbit TNF are explored further.

### MATERIALS AND METHODS

*TNF production.*—TNF was obtained from rabbits given 2 i.v. injections, 2 weeks apart, of BCG ($50–250 \times 10^6$ organisms) and endotoxin (100 $\mu$g). The animals were bled 2 h after the endotoxin injection; the recovered serum being designated "TNF serum".

*TNF assay.*—TNF was assayed by cytotoxicity *in vitro* against the mouse L929 cell line as reported previously (Matthews, 1979). Briefly, serum dilutions were incubated with L cells in flat-bottomed microtrays for 3 days at 37°C. The number of cells remaining was then quantitated photometrically after fixation and staining with Coomassie blue. The TNF titre is defined as the reciprocal of the dilution causing a 50% reduction in L cell numbers after 3 days of culture.

*Purification methods.*—The methods of ammonium sulphate precipitation, ion-exchange chromatography on DEAE-Sepharose and gel filtration on Ultrogel AcA44 have been described previously (Matthews & Watkins, 1978; Matthews, 1978). Fractions were assayed for TNF titre and also monitored for contaminating serum protein by "fused rocket" electroimmunoassay (Rose & Harboe, 1970) with a polyvalent sheep antiserum to rabbit serum. TNF-containing fractions were pooled to minimize contamination with other serum proteins.

*Polyacrylamide gel electrophoresis.*—Gradient, 8–36% polyacrylamide gels (Margolis &

Correspondence to: Dr N. Matthews, Department of Medical Microbiology, Welsh National School of Medicine, Heath Park, Cardiff CF4 4XN.

Kenrick, 1968) were run in a Pharmacia GE-4 tank and stained for protein with Coomassie blue (Fehrnström & Moberg, 1977) or Amido black, or for carbohydrate with the periodic acid–Schiff procedure. Samples were always run in duplicate in adjacent tracks; one track being stained and the other sliced into 20–30 portions for tests of TNF activity. Proteins were extracted from the gel as described previously (Matthews, 1978). Mol.-wt standards were bovine serum albumin, monomer and dimer (mol. wts 67,000 and 134,000), ovalbumin (mol. wt 43,000) and soya bean trypsin inhibitor (mol. wt 20,000).

*Isoelectric focusing.*—Isoelectric focusing was performed either in polyacrylamide gel with an LKB Ampholine PAG plate (pH range 3·5–9·5) in an LKB 2117 Multiphor tank (Winter *et al.*, 1977) or in a flat-bed granulated gel (LKB Ultrodex) with a pH range 4·0–6·0 (Winter *et al.*, 1975).

*Enzyme digestions.*—Trypsin (type III), papain (type III) and pronase (type VI) were purchased from Sigma. TNF, partially purified by ammonium sulphate precipitation and ion-exchange chromatography was diluted in the appropriate buffers: 46mM tris-HCl, 12mM $CaCl_2$ (pH 8·1) for trypsin; 25mM tris-HCl, 40mM EDTA, 10mM cysteine for papain; 80mM tris-HCl, 100mM $CaCl_2$ (pH 7·8) for pronase. Trypsin or pronase were added to give 1 mg/ml final concentration and papain to give a 1/3000 dilution, and the mixtures were incubated for 24 h at 37°C.

*Isopycnic ultracentrifugation.*—Five ml TNF serum, diluted 1/100 in CsCl solution (density = 1·406) was centrifuged at 42,000 rev/min for 40 h in a Beckman SW 50 rotor. Fractions (0·25 ml) were collected using an Isco density-gradient fractionator. Each fraction was assayed for refractive index (using an Abbé refractometer), TNF activity and albumin concentration (using "rocket" electroimmunoassay).

*Lectin chromatography.*—Lectins were prepared as follows: concanavalin A (Agraval & Goldstein, 1967), lentil lectin (Sage & Green, 1972—"method B"), ricin agglutinin, $RCA_{120}$ (Nicolson & Blaustein, 1972), soya-bean and wheat-germ (Vretblad, 1976), peanut agglutinin (Lotan & Sharon, 1978). The lectins were attached to CNBr-activated Sepharose 4B in the presence of the appropriate inhibitory monosaccharide. At room temperature, 1 ml of 1/10 TNF serum was run through a 2ml Sepharose lectin column equilibrated with isotonic phosphate-buffered saline (pH 7·2) (PBS); the con A column buffer also included 1mM $Ca^{+2}$, $Mg^{+2}$ and $Mn^{+2}$. The unabsorbed material was eluted in 10 ml of PBS, and the absorbed material in 10 ml of the appropriate saccharide in PBS. Both fractions were dialysed against PBS before testing. The saccharides used to elute absorbed material were 0·2M methyl pyranoside (Con A and lentil), 0·2M lactose (soya bean and peanut), 0·2M galactose (ricin) and 10% (w/v) N-acetyl glucosamine (wheat germ).

### RESULTS

### *Purification and molecular weight*

TNF was purified sequentially by precipitation with 50% saturated ammonium sulphate, ion-exchange chromatography on DEAE–Sepharose (Fig. 1) and gel-filtration on Ultrogel AcA44 (Fig. 2). Often, at this stage the TNF preparation contained 1 or 2 contaminating serum proteins detectable by rocket electroimmunoassay which could be removed by passage through a Con A column without significant loss of TNF activity. The final TNF preparations were purified about 1000-fold and contained no rabbit serum proteins detectable by electroimmunoassay. The overall recovery was 5–10%.

TNF serum on gradient PAGE electrophoresis gave a single peak of activity with a mol. wt of 67,000 (Fig. 3). TNF purified as above was eluted in a similar position, though in parallel stained gels, with purified TNF at 2–4 × serum concentration, no protein or carbohydrate-staining lines were visible either in the corresponding region or elsewhere in the gel, even after the application of large sample volumes to the gel.

Despite the mol. wt of 67,000 determined by gradient PAGE electrophoresis, TNF was regularly eluted as a single peak of apparent mol.-wt 39,000 on Ultrogel AcA44 (Fig. 2).

### *Isoelectric point*

The TNF activity of TNF serum was found in the pI range of 4·9–5·4 after isoelectric focusing in polyacrylamide gel

FIG. 1.—Fractionation by ion-exchange chromatography on a DEAE-Sepharose column (1·5 x 36 cm) of the TNF-rich fraction after precipitation with 50% saturated ammonium sulphate. The flow rate was 25 ml/h and 8·3 ml fractions were collected.

FIG. 2.—Fractionation by gel filtration on an Ultrogel AcA44 column (1·5 x 74 cm) of the TNF-rich fraction from the DEAE-Sepharose column. The flow rate was 7·2 ml/h and 2·4 ml fractions were collected.

FIG. 3.—Gradient polyacrylamide-gel electrophoresis of TNF serum. Two tracks were run in parallel, one being stained with Coomassie blue (upper) and the second being chopped into 30 fractions for testing TNF activity. The arrows indicate the elution positions of the mol. wt markers, bovine albumin (Alb), albumin dimer ($Alb_2$), ovalbumin (Ova) and soya bean trypsin inhibitor (TI).

FIG. 4.—Isoelectric focusing of TNF serum on an Ultrodex matrix with an ampholine range of pH 4–6.

FIG. 5.—Susceptibility of TNF to the proteinase pronase. Control TNF (▲), pronase-treated TNF (▼), pronase alone ( x ).

with an ampholine range of pH 3·5–9·5. Using an Ultrodex matrix with an ampholine range of pH 4–6 a pI value of 5·1–5·2 was obtained (Fig. 4).

*Chemical nature*

TNF, partially purified by salt precipitation and ion-exchange chromatography, was resistant to digestion for 24 h with the proteinases trypsin or papain, but susceptible to the less specific proteinase, pronase (Fig. 5). Resistance to trypsin and papain was not due to contamination of the TNF preparation with serum enzyme inhibitors, as in both instances proteolytic-enzyme activity was still in the digestion mixture after incubation.

On isopycnic ultracentrifugation TNF had a buoyant density of 1·27 (Fig. 6) confirming its protein nature with little or no carbohydrate.

TNF failed to bind to peanut agglutinin, soya bean and ricin lectins, and $<5\%$ of the activity was retained by concanavalin A, lentil lectin or wheat-germ agglutinin columns (Fig. 7). Thus, if a carbohydrate side chain is present, most of the TNF molecules lack mannose, glucose, glucosamine, galactose or galactosamine end groups.

Rabbit TNF was stable to heating for 20 min at 56°C or 70°C, but completely destroyed by heating at 100°C for 20 min.

DISCUSSION

Fractionation of TNF serum, either by gel-filtration, isoelectric focusing or gradient PAGE, gave a single peak of activity indicating that rabbit TNF is a single molecular species. The susceptibility to pronase, buoyant density of 1·27 and lack of binding to a range of lectins suggests that rabbit TNF is largely protein.

In this study, column fractions were tested for TNF at a range of dilutions, and

FIG. 6.—Isopycnic ultracentrifugation of TNF serum.

FIG. 7.—Binding of TNF to lectin columns. (a) control TNF (▲), peanut agglutinin-unbound (□) and bound (■), con A-unbound (▽) and bound (▼). (b) control TNF (▲), lentil lectin-unbound (□) and bound (■), soya bean lectin-unbound (▽) and bound (▼). (c) control TNF (▲), wheatgerm lectin-unbound (□) and bound (■), ricin lectin-unbound (▽).

the activity expressed as a titre rather than cytoxocity at a single concentration. As a result the gel-filtration profile is more homogeneous than shown previously (Matthews & Watkins, 1978). However, the mol. wt of 39,000 obtained by gel-filtration differs significantly from the gradient PAGE determination of 67,000. The most likely explanation is that TNF is retarded on gel-filtration by interaction with the gel-matrix. Mol. wt determination using SDS-PAGE was not possible because firstly, TNF activity was irreversibly lost after SDS treatment and secondly, the most purified preparations of TNF failed to stain with protein or carbohydrate stains after electrophoresis.

The purification procedure adopted here regularly gave TNF preparations which were purified about 1000-fold, and uncontaminated with serum proteins as revealed by electroimmunoassay. However, purified TNF preparations failed to give protein-staining bands after gel-electrophoresis. Thus, either TNF is poorly reactive with protein stains, or it comprises only a part of the "purified" TNF preparations; the remainder being a mixture of contaminating serum proteins, each present in too small an amount to give a distinct line after gel-electrophoresis. Until very recently, purification of interferon had reached a similar stage (see Stewart, 1977). Methods analogous to those now used for interferon purification (specific physical or immuno-adsorbents) may be necessary to purify TNF completely.

This work was supported by a grant from the Cancer Research Campaign.

### REFERENCES

AGRAVAL, B. B. L. & GOLDSTEIN, I. J. (1967) Protein carbohydrate interaction—VI. Isolation of Concanavalin A by specific adsorption on cross-linked dextran gel. *Biochim. Biophys. Acta*, **147**, 262.

CARSWELL, E. A., OLD, L. J., KASSEL, R. L., GREEN, S., FIORE, N. & WILLIAMSON, B. (1975) An endotoxin-induced serum factor that causes necrosis of tumors. *Proc. Natl Acad. Sci. U.S.A.*, **72**, 3666.

FEHRNSTRÖM, H. & MOBERG, U. (1977) SDS and conventional polyacrylamide gel electrophoresis with LKB 2117 Multiphor. *LKB Application Note* 306.

GREEN, S., DOBRJANSKY, A., CARSWELL, E. A. & 4 others (1976) Partial purification of a serum factor that causes necrosis of tumors. *Proc. Natl Acad. Sci. U.S.A.*, **73**, 381.

LOTAN, R. & SHARON, N. (1978) Peanut (*Arachis hypogae*) agglutinin. In *Methods in Enzymology*, Vol. 50. Ed. Ginsburg. New York: Academic Press. p. 361.

MARGOLIS, J. & KENRICK, K. G. (1968) Polyacrylamide gel electrophoresis in a continuous molecular sieve gradient. *Anal. Biochem.*, **25**, 347.

MATTHEWS, N. (1978) Tumour necrosis factor from the rabbit. II. Production by monocytes. *Br. J. Cancer*, **38**, 310.

MATTHEWS, N. (1979) Tumour necrosis factor from the rabbit. III. Relationship to interferons. *Br. J. Cancer*, **40**, 534.

MATTHEWS, N. & WATKINS, J. F. (1978) Tumour necrosis factor from the rabbit. I. Mode of action, specificity and physicochemical properties. *Br. J. Cancer*, **38**, 302.

NICOLSON, G. L. & BLAUSTEIN, J. (1972) The interaction of *Ricinus* agglutinin with normal and tumor cell surfaces. *Biochim. Biophys. Acta*, **266**, 543.

ROSE, C. & HARBOE, N. M. G. (1970) Preparative isoelectric focusing in a sea-serpent shaped apparatus. In *Protides of the Biological Fluids*, Vol. 17. Ed. Peeters. Oxford, Pergamon Press. p. 397.

SAGE, M. J. & GREEN, R. W. (1972) Common lentil (*Lens culinaris*) phytohaemagglutinin. In *Methods in Enzymology*, Vol. 28. Ed. Ginsburg. New York: Academic Press. p. 332.

STEWART, W. E. II (1977) Purification and characterization of interferons. In *Interferons and their Actions*. Ed. Stewart. Ohio: CRC Press Inc. p. 49.

VRETBLAD, P. (1976) Purification of lectins by biospecific affinity chromatography. *Biochim. Biophys. Acta*, **434**, 169.

WINTER, A., EK, K. & ANDERSSON, U.-B. (1977) Analytical electro-focusing in thin layers of polyacrylamide gels. *LKB Application Note* 250.

WINTER, A., PERLMUTTER, H. & DAVIES, M. (1975) Preparative flat-bed electrofocusing in a granulated gel with the LKB 2117 Multiphor. *LKB Application Note* 198.


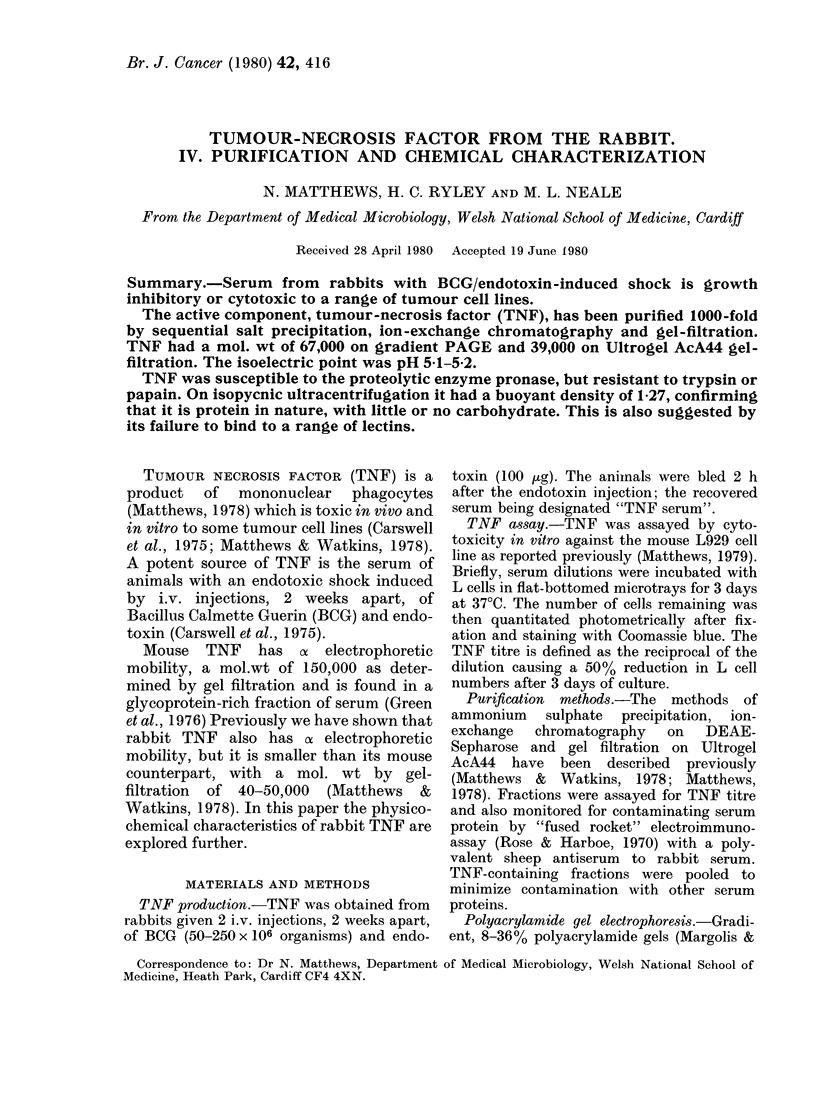

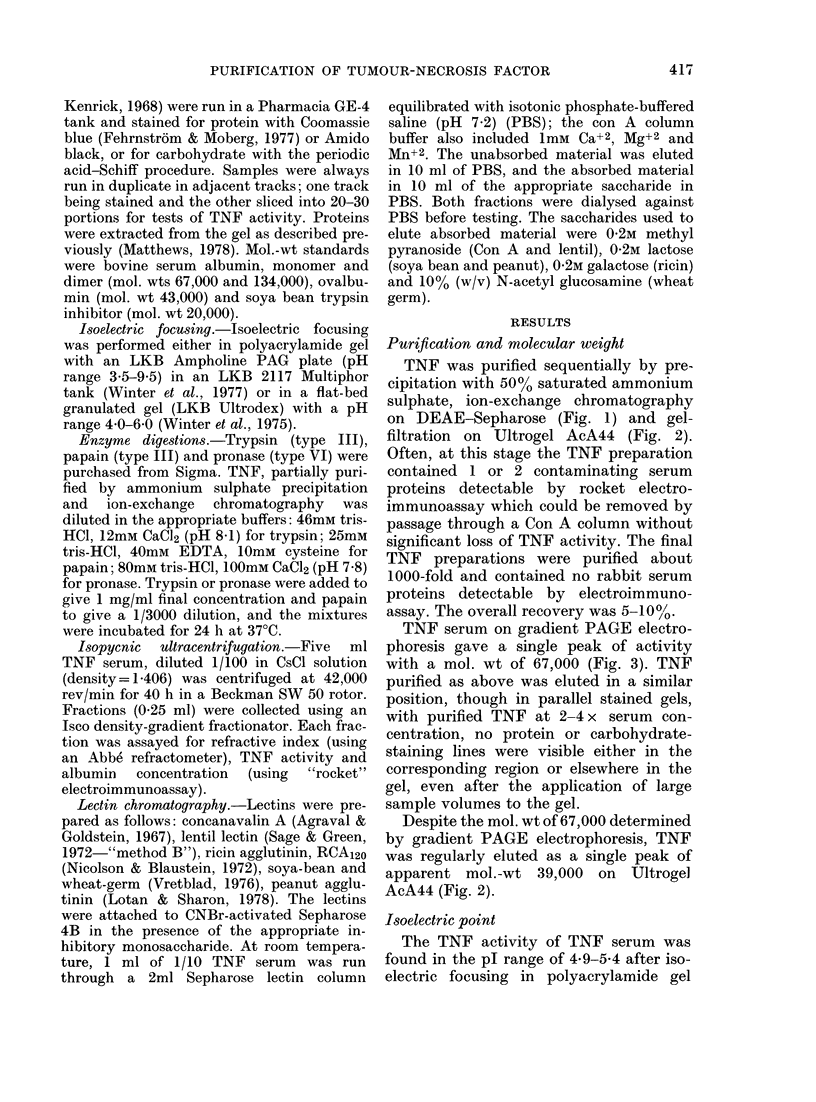

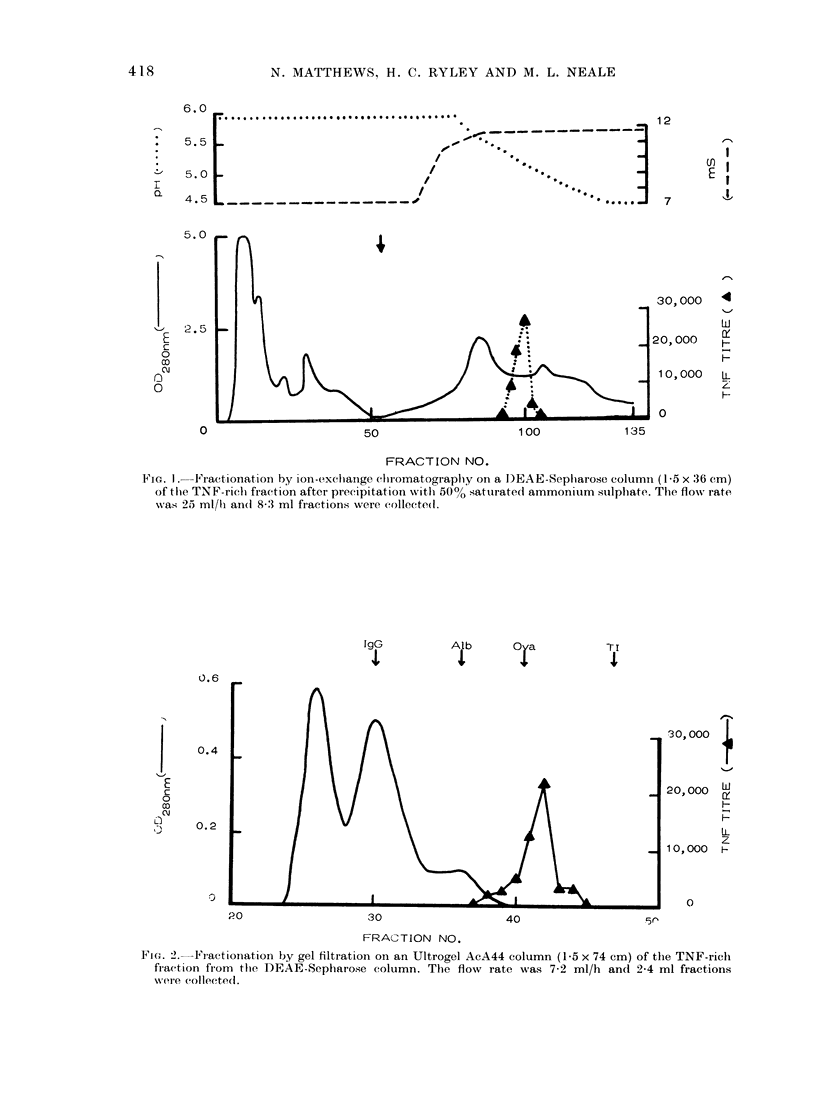

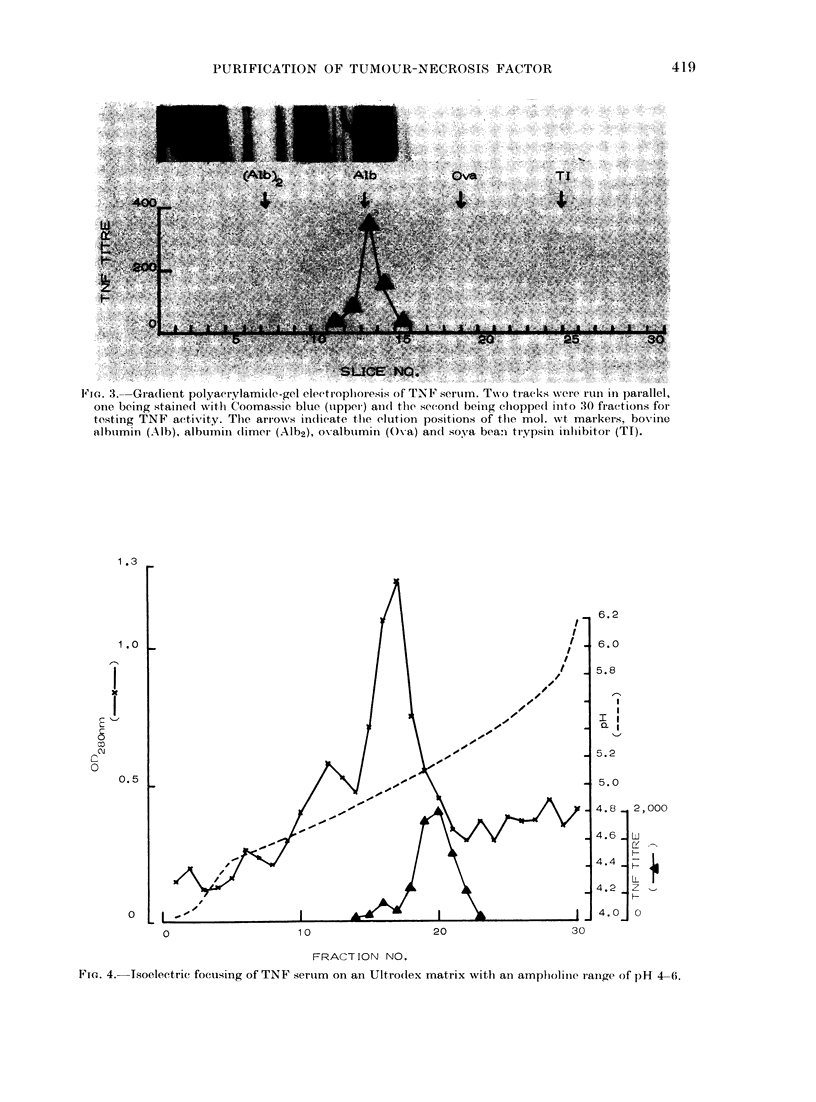

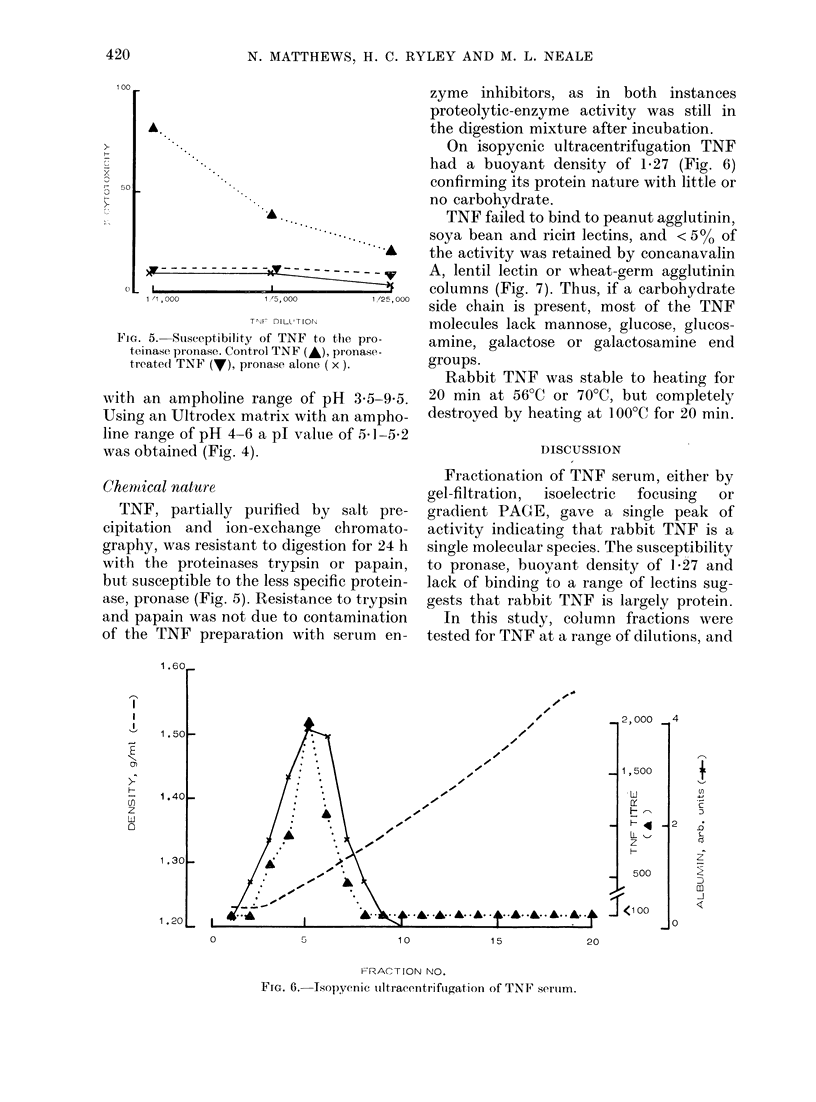

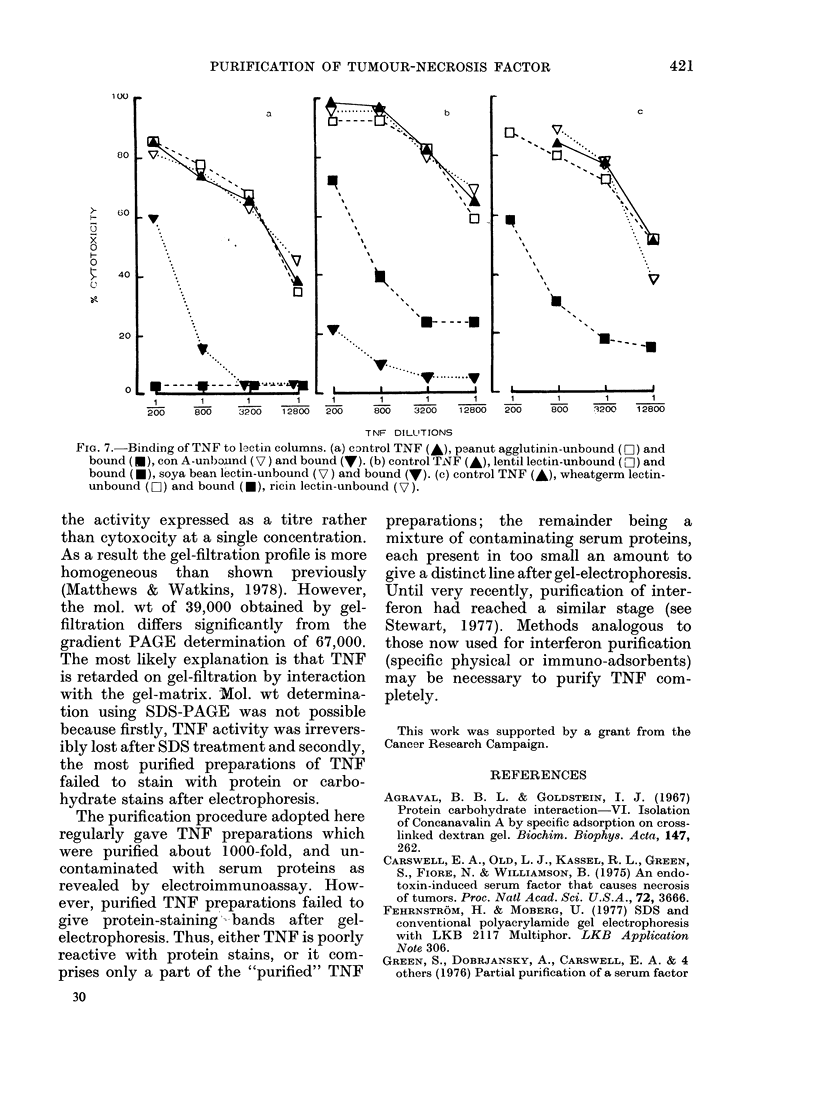

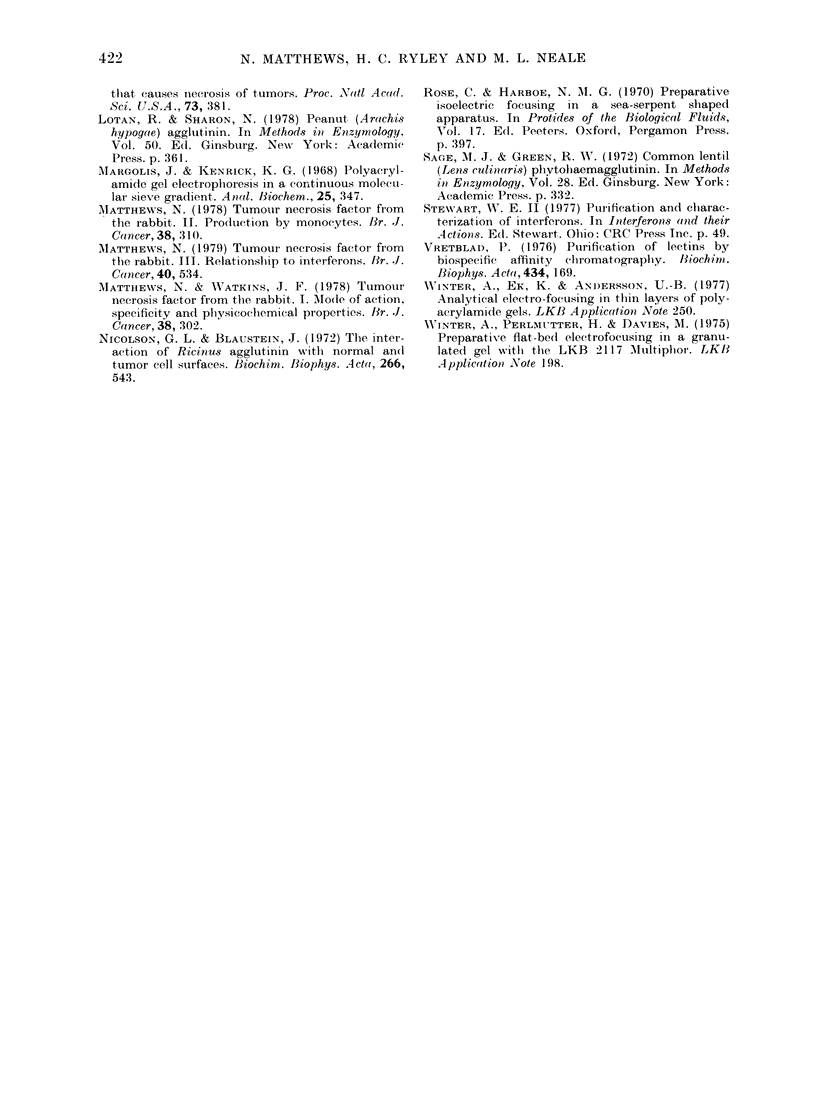


## References

[OCR_00579] Agrawal B. B., Goldstein I. J. (1967). Protein-carbohydrate interaction. VI. Isolation of concanavalin A by specific adsorption on cross-linked dextran gels.. Biochim Biophys Acta.

[OCR_00586] Carswell E. A., Old L. J., Kassel R. L., Green S., Fiore N., Williamson B. (1975). An endotoxin-induced serum factor that causes necrosis of tumors.. Proc Natl Acad Sci U S A.

[OCR_00608] Lotan R., Sharon N. (1978). Peanut (Arachis hypogaea) agglutinin.. Methods Enzymol.

[OCR_00614] Margolis J., Kenrick K. G. (1968). Polyacrylamide gel electrophoresis in a continuous molecular sieve gradient.. Anal Biochem.

[OCR_00624] Matthews N. (1979). Tumour-necrosis factor from the rabbit. III. Relationship to interferons.. Br J Cancer.

[OCR_00629] Matthews N., Watkins J. F. (1978). Tumour-necrosis factor from the rabbit. I. Mode of action, specificity and physicochemical properties.. Br J Cancer.

[OCR_00637] Nicolson G. L., Blaustein J. (1972). The interaction of Ricinus communis agglutinin with normal and tumor cell surfaces.. Biochim Biophys Acta.

[OCR_00658] Vretblad P. (1976). Purification of lectins by biospecific affinity chromatography.. Biochim Biophys Acta.

